# A new era of stem cell and developmental biology: from blastoids to synthetic embryos and beyond

**DOI:** 10.1038/s12276-023-01097-8

**Published:** 2023-10-02

**Authors:** Yunhee Kim, Inha Kim, Kunyoo Shin

**Affiliations:** 1 School of Biological Sciences, College of Natural Sciences, Seoul National University, Seoul, 08826 Republic of Korea; 2 Institute of Molecular Biology and Genetics, Seoul National University, Seoul, 08826 Republic of Korea

**Keywords:** Stem cells, Organogenesis, Pattern formation, Embryogenesis, Disease model

## Abstract

Recent discoveries in stem cell and developmental biology have introduced a new era marked by the generation of in vitro models that recapitulate early mammalian development, providing unprecedented opportunities for extensive research in embryogenesis. Here, we present an overview of current techniques that model early mammalian embryogenesis, specifically noting models created from stem cells derived from two significant species: *Homo sapiens*, for its high relevance, and *Mus musculus*, a historically common and technically advanced model organism. We aim to provide a holistic understanding of these in vitro models by tracing the historical background of the progress made in stem cell biology and discussing the fundamental underlying principles. At each developmental stage, we present corresponding in vitro models that recapitulate the in vivo embryo and further discuss how these models may be used to model diseases. Through a discussion of these models as well as their potential applications and future challenges, we hope to demonstrate how these innovative advances in stem cell research may be further developed to actualize a model to be used in clinical practice.

## Revolutionizing embryogenesis: new stem cell discoveries

Researchers create synthetic embryos, advancing our understanding of early human development and offering new possibilities for disease modeling. In a significant breakthrough, scientists have generated synthetic embryos using stem cells, providing a more accurate model of early human development. These models, including blastoids and gastruloids, mimic the structure and function of early embryos and can be used to study the molecular mechanisms governing embryogenesis. By replicating various stages of development, synthetic embryo models can help researchers investigate congenital diseases and potential clinical applications. As this technology continues to evolve, it may lead to new insights into human biology and the development of novel therapies for a range of conditions.

## Introduction

The 1995 Nobel Prize in Physiology or Medicine, awarded to Edward B. Lewis, Christiane Nüsslein-Volhard, and Eric F. Wieschaus, marked a turning point in the field of modern developmental biology. By introducing mutations in *Drosophila* embryos, these researchers found key genes associated with regulating several significant early developmental processes, such as segmentation and polarization in *Drosophila*. Their groundbreaking discoveries in the genetic control of embryonic development laid the foundation for a deeper understanding of the molecular mechanisms governing the formation of multicellular organisms^1,2^.

Over the past three decades, substantial progress has been made in developmental biology ever since the foundation of several techniques, such as genetic engineering techniques, wherein various animal model systems were actively utilized to further enhance the understanding of genetic functions within the processes of embryonic development. However, studying human development has remained a significant challenge due to the inherent differences between conventional model organisms and humans^3^, ethical concerns about using human embryos^4^, and physical inaccessibility due to the intrauterine development of in vivo human embryos.

Recently, the rapid advancement of stem cell research has enabled the generation of stem cell-derived human organoids^5^, which are three-dimensional, self-organizing structures that mimic various human organs. This remarkable progress has expanded the boundaries of human biology research, allowing the investigation of human biological processes in a more physiologically relevant context^6^. In addition to organoids, recent advances in stem cell research have even facilitated the development of early embryonic models that mimic human embryos. Early embryonic models provide an opportunity to investigate molecular mechanisms that regulate human embryogenesis with better scalability and in a relatively ethical manner, thereby overcoming the major limitations of using natural human embryos^7–10^.

Early embryonic models were generated by using embryonic stem cells (ESCs)^11^, which possess the ability to self-renew and differentiate into a variety of specific cell types of the body. The potency of ESCs varies depending on their epigenetic traits and culture environment^12^. These shifts in potency allow the induction of various in vitro models, including synthetic whole embryo models that incorporate all significant cell types, such as those derived from both the embryo and extraembryonic tissues^8^. This review describes the process by which stem cells, including ESCs in relation to their potency, are utilized to create these in vitro models.

This review aims to explore developments in stem cell research, focusing on stem cell-based in vitro early embryonic developmental models. Overall, this review article aims to provide a comprehensive analysis of the new era of stem cell research, examining the potential uses of developmental-stage-specific embryo models for disease modeling and possible clinical applications. By providing a thorough discussion of these technologies, we hope to inspire further research and advancements in the field.

## Embryonic stem cells (ESCs): the building blocks of synthetic embryos

**Fig. 1:**
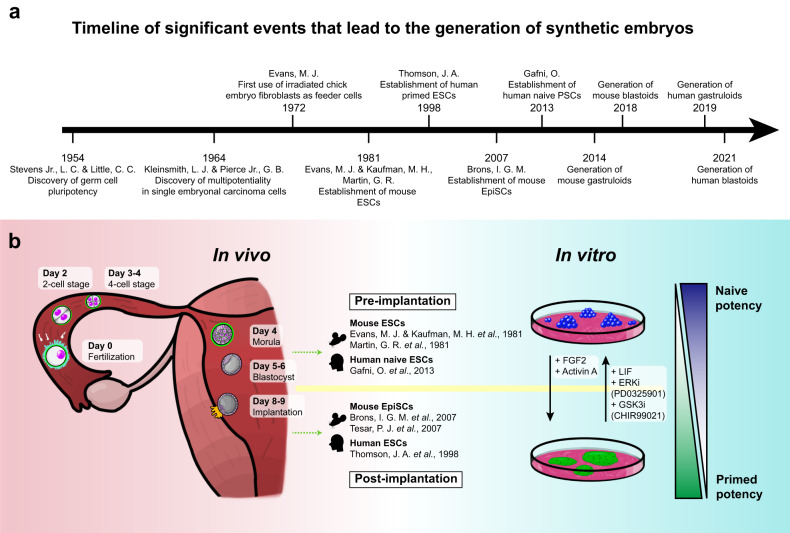
Timeline of seminal events in the fields of stem cell and developmental biology and in vivo embryogenesis including in vitro counterparts. **a** Timeline of significant events in stem cell research facilitating the generation of synthetic embryos. **b** Illustration of early mammalian development in vivo (left), as well as the in vitro counterparts—naïve and primed ES cells—represented as cell states (right). Each developmental stage of the mammalian embryo is depicted from the fertilization of the egg (Day 0) to the implantation of the blastocyst (Day 9). Pre- (top) and post-implantation (bottom) stem cells derived in vitro are listed in the middle, as well as the original research in which they were discovered. The ESCs that correspond to the pre- and post-implantation states are naïve (top) and primed (bottom) ESCs, respectively, and the degrees of the states are represented on the right (ascending, more naïve; descending, more primed). Morphogens that regulate their states are written in between.

Stem cells are self-renewing cells with the potential to differentiate into a wide variety of cell types^13^. Based on their origin, they are divided into two major categories: ESCs and adult stem cells (AdSCs). ESCs are derived from the inner cell mass (ICM) of blastocysts and can differentiate into any type of cell in the body^14^, whereas AdSCs are found in various tissues in our body and help maintain tissue homeostasis but can differentiate into only a limited number of cell types relative to ESCs^13,15^. Early embryonic in vitro models were all derived from ESCs or induced pluripotent stem cells (iPSCs)^16^ of equivalent potency. This section focuses on ESCs, which serve as the foundation for creating in vitro models, and analyzes their diverse potencies (Fig. 1a).

### Birth of ESCs

The concept of ESCs was first proposed in the 1950s by Stevens and Little, who discovered that spontaneous testicular teratomas in an inbred mouse strain originated from primordial germ cells and that these cells can differentiate into a variety of cell types^17^. This discovery illuminated the pluripotency of germ cells, opening the way for further investigation into the origin and differentiation potential of other stem cell types. Kleinsmith and Pierce expanded upon these findings by demonstrating the multipotentiality of single embryonal carcinoma cells^18^.

The development of in vitro culture methods also played a crucial role in the establishment of ESCs. Evans pioneered the use of irradiated chick embryo fibroblasts as feeder cells to culture mammalian stem cells in vitro, which enabled the maintenance and expansion of pluripotent stem cells (PSCs)^19^. Based on these findings, Evans, Kaufman, and Martin successfully derived mouse ESCs (mESCs) from the ICM of blastocysts, marking the beginning of modern stem cell research^20,21^. Building on the advancements in mESC research, the scientific focus shifted to human ESCs (hESCs), leading to a significant breakthrough in 1998 when Thomson et al. successfully derived and cultured hESCs in vitro by isolating them from the ICM of human blastocysts^14^. Both mouse and human ESCs possess the capacity for self-renewal and pluripotency, which allows them to maintain an undifferentiated state while proliferating in culture and to differentiate into any cell type of the three germ layers (endoderm, mesoderm, and ectoderm)^14,20^.

Although initially established mESCs and hESCs share certain traits, they also display distinct differences in morphological, transcriptional, and epigenetic features^22^. mESCs, for instance, have a tendency to form a dome-like structure, whereas hESCs typically exhibit a flattened shape^22^. Both mESCs and hESCs express the pluripotency-related transcription factor octamer-binding transcription factor 4 (OCT4), albeit with distinct regulatory mechanisms. In mESCs, OCT4 expression is regulated by its distal enhancer, while in hESCs, its expression is mainly regulated by its proximal enhancer^23,24^.

As subsequent research progressed, a method was developed to culture mESCs that exhibited characteristics similar to hESCs^25,26^. These cells, derived from the epiblast of post-implantation embryos, were termed mouse epiblast stem cells (mEpiSCs) and shared morphology and a variety of epigenetic properties similar to hESCs^26^. Continuous investigations have revealed that the differences between mESCs and hESCs originate from the fact that these ESCs represent different stages of development, thereby influencing their slightly different differentiation potentials.

### Naïve and primed pluripotent states of ESCs

Mammalian ESCs are known to exist in a continuum of configurations, which at either end of the spectrum lie two different pluripotent states: naïve and primed^12^. These states have distinct molecular and functional characteristics, and understanding their differences is crucial for optimizing the use of ESCs in modeling early embryos in vitro.

Naïve ESCs, compared to primed ESCs, correspond to an earlier developmental stage of pluripotent cells. They are derived from the pre-implantation ICM during the early blastocyst stage^27^ (Fig. 1b). These cells exhibit characteristics such as having a relatively unrestricted differentiation potential, allowing them to differentiate into both embryonic (epiblast) and extraembryonic tissue (hypoblast). In addition, they have a more open chromatin structure on developmental regulatory gene promoters, accompanied by a global reduction in DNA methylation^27,28^.

In contrast, primed ESCs are derived from a developmentally more advanced stage, specifically from post-implantation epiblast cells^27^ (Fig. 1b). These cells exhibit a relatively restricted differentiation potential; they predominantly contribute to embryonic components, unlike their naïve counterparts^25^. This restriction is likely due to their relatively closed chromatin structure on lineage regulatory genes^29^.

The first mESCs established were in the naïve state^20,21^, while the initial hESCs and mEpiSCs that were established resembled the primed state of pluripotency^14,25^. Over several years, efforts to establish human naïve ESCs have led researchers to gain insights into the molecular mechanisms and signaling pathways regulating pluripotency. These key insights included the identification of factors essential for maintaining naïve pluripotency in mESCs, such as leukemia inhibitory factor (LIF), which activates the Janus kinase-signal transducer and activator of transcription 3 (JAK-STAT3) signaling pathway^30,31^. The development of specific culture conditions to derive and maintain mEpiSCs facilitated further investigation into the signaling pathways that regulate primed pluripotency, including the fibroblast growth factor/extracellular signal-regulated kinase (FGF/ERK) and Activin/Nodal pathways^25,26^.

The establishment of naïve hESCs required the identification of culture conditions that could support the naïve state while suppressing the primed state. This requirement led researchers to focus on modulating signaling pathways, including the inhibition of the FGF/ERK^32^ and transforming growth factor-beta (TGF-β)/Activin/Nodal pathways^33^, which are known for maintaining primed pluripotency. The application of small molecules to target these pathways enabled the conversion of primed hESCs into a state resembling naïve pluripotency^23,34^.

After years of extensive efforts, the landmark achievement of establishing naïve hESCs was made by Gafni et al. in 2013. Gafni and colleagues successfully established naïve hESCs using a combination of small molecules and growth factors^35^. They showed that inhibiting the FGF/ERK, TGF-β, and WNT/β-catenin pathways while simultaneously activating the LIF/STAT3 signaling pathway could prompt the conversion of primed hESCs to a naïve state^36^. These newly established naïve hESCs exhibited key features of naïve pluripotency, including the acquisition of epigenetic states similar to those of naïve mESCs, the expression of naïve-specific markers, and the attainment of an enhanced differentiation potential^23,35^.

## Blastoids modeling early embryo development

The early stages of mammalian embryogenesis, leading up to implantation, encompass a series of conserved, systematic steps across various species, including ovulation, fertilization, cleavage, and implantation. Upon ovulation, the oocyte travels through the oviduct, where fertilization takes place in the ampulla. As the embryo travels toward the uterus, it undergoes asynchronous cleavages. At this juncture, the mammalian genome is activated, facilitating embryonic development through the expression of early transcribed proteins.

In humans, major waves of zygotic gene activation occur at the 8-cell stage, whereas in mice, activation arises at the 2-cell stage (minor waves transpire at the 4-cell stage and late zygotic stage for human and mouse embryos, respectively)^37–40^. Researchers have endeavored to model this process by generating human eight-cell-like cells (8CLCs) to probe the mechanism of totipotency^41^. In 2022, Mazid et al. developed a method to produce 8CLCs from human PSCs (hPSCs), revealing key roles of developmental pluripotency associated 3 (DPPA3), a master regulator of DNA methylation in oocytes, and tetrapeptide repeat homeobox 1 (TPRX1), a eutherian totipotent cell homeobox (ETCHbox) family transcription factor, in this process. Using this embryo model, the researchers demonstrated that 8CLCs can contribute to embryonic and extraembryonic lineages, providing a valuable resource for the study of the earliest stages of human embryogenesis.

Compaction is one of the most significant events of mammalian cleavage. In mice, after the embryo reaches the 8-cell stage, compaction occurs as blastomeres express cell adhesion proteins and coalesce into a sphere of cells^42^. This compact embryo matures into a 16-cell morula, where its inner cells later become constituents of the ICM, and its outer cells give rise to the trophectoderm (TE)^43^. The ICM is situated on one side within the TE, culminating in the formation of the blastocyst. In essence, the blastocyst comprises two primary cell populations: the ICM and the TE.

**Fig. 2:**
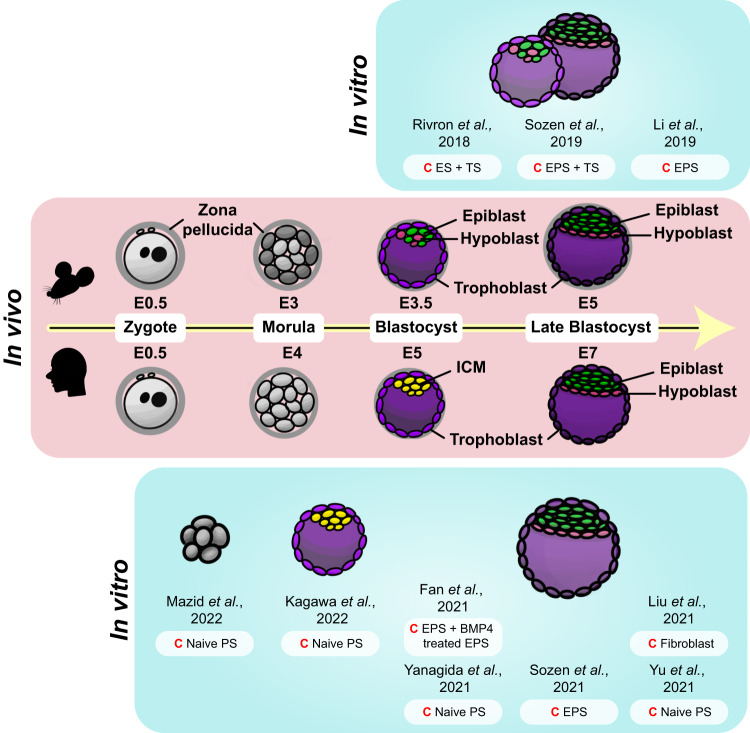
Schematic representation of the in vivo embryo and corresponding in vitro models of the pre- and peri-implantation stages. A depiction of the early developmental stages, spanning from the zygote phase (E0.5 of both mouse and human embryo) to the blastocyst stage (E5 and E7 of the mouse and human embryo, respectively), of mouse and human embryos are portrayed in the middle. In vitro models corresponding to the in vivo embryo of each developmental stage are illustrated on either side (top, mouse; bottom, human). Specific cells are color-coded throughout the figure (epiblast, green; hypoblast, pink; trophoblast, purple; ICM, yellow); the same color represents similar states at which they are in. Starting cells (C) of each model are noted adjacent to each illustration.

The peri-implantation period designates the phase during which the blastocyst is unattached in the uterus, preceding its implantation into the uterine wall, and differentiates into several fundamental structures. The nascent cells of the ICM segregate to form the primitive endoderm (PrE) and the epiblast. These intricate processes, which are fundamental to early embryonic development, have historically been difficult to study in depth. However, recent advances in stem cell research have now made it possible to create in vitro models that closely mimic these early stages of development (Fig. 2).

Since the isolation of mESCs in the 1980s^20,21^, stem cell research has significantly advanced. With the subsequent discovery of trophoblast stem cells (TSCs)^44^ and extraembryonic endoderm stem cells (XENs)^45^, researchers have been equipped with essential cellular tools for the creation of in vitro mouse embryo-like structures known as mouse blastoids. This marked a pivotal breakthrough in embryonic research, signifying the first successful attempt to replicate an entire embryo in vitro.

Rivron et al. set a precedent by successfully generating the first mouse blastoids^46^. Following this breakthrough, numerous research groups have also developed similar in vitro structures that mimic in vivo blastocysts by employing a variety of methods^46–48^. Mouse blastoids are generated by aggregating various types of stem cells, including pluripotent and extraembryonic stem cells, in non-adherent hydrogel microwells, utilizing the self-organizing property of the mammalian embryo^43^. These cells are cultured with a blend of morphogens that influence the specification of the epiblast and TE lineages. Through this self-assembly process, in vitro structures that strikingly resemble in vivo blastocysts are created.

A pivotal event in successful blastoid formation is cavitation, underpinned by TE formation. TE formation is fostered by inhibiting the Hippo and TGF-β pathways. Concurrently, the addition of LIF and inhibitors of mitogen-activated protein kinase kinase (MEK) and glycogen synthase kinase 3 (GSK3) (known as 2i) helps maintain the pluripotency of ESCs by activating the STAT3, ERK, and WNT signaling pathways^49^. Moreover, Y-27632, a Rho-associated protein kinase (ROCK) inhibitor, is utilized to prevent cell apoptosis during this complex process.

In just 3 to 4 days, blastoids successfully take shape, exhibiting features such as the blastocoel cavity, ICM, and TE layer, the organization of which closely replicates the spatial organization seen in in vivo blastocysts. As cells start the process of self-assembly into blastoids, additional transcription factors, including GATA binding protein 6 (GATA6) and caudal type homeobox 2 (CDX2), are activated. These transcription factors govern lineage specification and cell differentiation, with GATA6 promoting the formation of the PrE lineage and CDX2 playing a crucial role in inducing the proper differentiation of the TE lineage^43,50,51^. The precise activation of transcription factors within blastoids is essential for directing the self-organization and lineage segregation of ESCs and TSCs into a fully functional blastoid structure. These blastoids share several characteristics with natural blastocysts, such as the capacity to acquire apical‒basal polarity and tight junctions.

Expanding upon strategies used in the creation of mouse blastoids, researchers have successfully generated human blastoids by harnessing the unique properties of hPSCs. Human naïve ESCs, notable for their increased plasticity and lower lineage barrier compared to naïve mESCs, have the capacity to differentiate into both embryonic (epiblast) and all extraembryonic (TE and hypoblast) lineages^52–54^. This significant capability allows the formation of human blastoids without necessitating the mixture of ESCs and TSCs^55–57^. In a parallel manner, human extended pluripotent stem cells (hEPSCs)^58,59^, cultured to embody developmental potency for embryonic and extraembryonic cell lineages, have shown the potential to generate blastoids^60^.

Several techniques have been developed to generate human blastoids. For instance, human blastoids can be generated by aggregating naïve hPSCs or hEPSCs in rounded microwells using centrifugation^55^, and similar results can be achieved with standard microwells or pyramid wells^56,57,60^. It should be noted that the use of a single cell type, such as naïve hPSCs or EPSCs, is not the only option to induce blastoid formation in the medium. Indeed, blastoids can also be generated through a combination of hEPSCs and TE-like cells derived from EPSCs^61^. Similarly, 8CLCs have been used to derive blastoids that exhibit both morphological and transcriptomic similarities to human blastocysts^41^.

Another breakthrough was the direct reprogramming of human somatic cells, which was achieved by infecting human dermal fibroblasts with a virus capable of inducing the expression of OCT4, SRY-box 2 (SOX2), Kruppel-like factor 4 (KLF4), and c-MYC, which in turn generated induced blastoids, or iBlastoids^62^. These human blastoids bear transcriptomic and morphological resemblances to human blastocysts, and remarkably, some have even shown the potential to initiate events akin to implantation.

Blastoids have the potential to be implanted into a hormone-stimulated endometrial layer^55^, allowing the observation of post-implantation processes. In compliance with the International Society for Stem Cell Research (ISSCR) guidelines^63^, which allow human experimental culture up to 14 days, these human blastoids could potentially be implanted into a synthetic endometrial lining for the purpose of investigating post-implantation events. The implantation of blastoids revealed that this process took place in the polar TE region, which likely became polarized due to its nascent relation to epiblast-like cells^55^. After implantation, sustained proliferation of epiblasts, TEs, and PrE-like cells was observed^55^.

Although several blastoids have cellular compositions similar to those of human in vivo blastocysts, it is necessary to investigate them more closely to determine whether they precisely replicate the in vivo state. The similarity of most of the blastoids created thus far to in vivo blastocysts has been validated using single-cell transcriptomics. Occasionally, however, the cells formed in blastoids do not match those of human early blastocysts but rather those of later developmental phases (such as gastrulation after embryonic day (E) 14)^64^. Therefore, further analysis is required to determine how accurately these in vitro models represent the in vivo context, and ongoing research should focus on increasing their similarity.

## Early gastruloids recapitulating the formation of three germ layers

Gastrulation is one of the most important processes of the developing mammalian embryo, where it incorporates several combinations of cell movements in newly positioning the embryonic tissue to form the three germ layers—ectoderm, mesoderm, and endoderm—which set a platform that further enables the navigation of cellular lineage specification by placing the tissues accordingly throughout the anterior-posterior (AP), dorsal-ventral, and lateral (left and right) axes. By appropriately positioning the embryonic tissue layers, organizing axes, and facilitating progressive specialization, the embryo is able to undergo processes such as organogenesis and limb morphogenesis that lead to the formation of more complex structures.

Going back to mark the initiation of the creation of synthetic embryos, as mentioned earlier, developmental biologists Martin and Evans found that ES-cell-like 2D clonal pluripotent teratocarcinoma cells suspended into aggregates self-organize themselves to acquire a cellular organization pattern similar to that of the differentiation of the early mouse embryo^65^. Following their discovery, other studies have likewise continued to generate 3D aggregates that were able to recapitulate early embryonic differentiation and thus were collectively termed “embryoid bodies (EBs)”. When EBs derived from ES cells of mouse blastocyst ICM origin were cultured under in vivo-like conditions with appropriate developmental cues, they acquired the ability to differentiate into all three germ layers^66,67^.

**Fig. 3:**
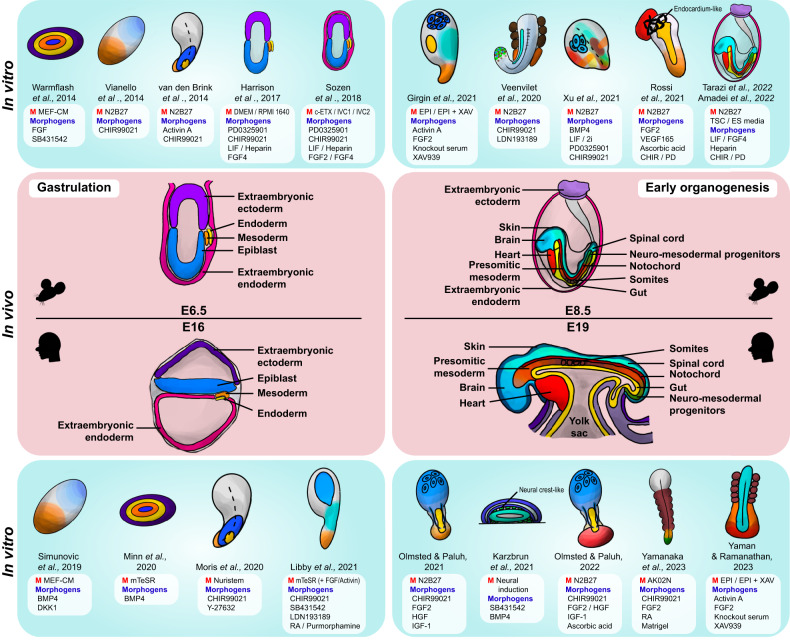
Schematic representation of the in vivo embryo and corresponding in vitro models of the post-implantation stages. In vivo developmental stages of the mouse and human embryos are depicted in the middle, from the process of gastrulation (E6.5 and E16 of the mouse and human embryo, respectively) to early organogenesis (E8.5 and E19 of the mouse and human embryo, respectively). In vitro models corresponding to the in vivo embryo of each developmental stage are illustrated on either side (top, mouse; bottom, human), as well as to note the original paper they were formed in. Specific tissues/organs are color-coded throughout the figure for both the embryos in gastrulation (mouse extraembryonic ectoderm, light purple; human extraembryonic ectoderm, dark purple; extraembryonic endoderm, pink; mesoderm, orange; endoderm, yellow; epiblast, blue) and those in early organogenesis (mouse extraembryonic ectoderm, light purple; extraembryonic endoderm, pink; brain, sky blue; spinal cord, turquoise; skin, dark navy; heart, red; notochord, dark red; somites, brown; presomitic mesoderm, dark orange; mesoderm, orange; gut and/or endoderm, yellow; neuromesodermal progenitors, green). Specific medium (M) or morphogens, including growth factors, cytokines, and inhibitors, used in the protocols for generating each in vitro model are listed adjacent to each gastruloid illustration.

Taking the potency of pluripotent stem cells to advantage, the addition of various signaling factors parallel to those that work at the corresponding developmental stage to EBs coaxed the formation of models that recapitulate the early development of the embryo. Due to the established role of signaling factors around this stage of embryonic development, small molecules added to induce the growth of in vitro models are short-range and similar among different protocols. Agonists of the FGF, BMP, and WNT signaling pathways (such as FGF2, BMP4, and CHIR99021, respectively)—key signaling pathways that govern development—as well as inhibitors (such as PD173074, LDN193189, and XAV939, respectively) are used to induce or inhibit characteristics of the developing embryo (Fig. 3).

As such, incorporating signaling pathways into in vitro cell aggregates yields models that recapitulate important hallmarks of gastrulation. For example, symmetry breaking is achievable in EBs in vitro, where the local activation of the WNT signaling pathway induces AP polarity and the formation of a primitive streak-like region, resulting in differentiation into a mesendodermal compartment (in contrast, neurectodermal differentiation is achieved by WNT signaling inhibition)^68^. Furthermore, the activation of WNT signaling at an appropriate time frame (at the initial stages of culture, which is usually within 74 h post-aggregation) induces the formation of a progressive elongating domain similar to that of the in vivo tail bud of the embryo, as well as the specification of the endoderm, which all lead to simulating aspects of the gastrulation period of the embryo^69^. Additionally, the precise timing regulated by WNT/β-catenin and Nodal signaling induces symmetry breaking in gastruloids even without the presence of extraembryonic tissue or localized signaling, allowing further polarization to take place, which is evidenced by distinct T/Brachyury expression^70^. Reducing FGF signaling yielded a diminished tail phenotype in the embryo models, indicating that the inducement of this signaling pathway is important for elongation^71^. With the knowledge of biological mechanisms underlying embryonic development and the use of key signaling factors, several models that mimic aspects of gastrulation were generated, starting from micropatterned 2D models (“2D gastruloids”), which encompass the endoderm, mesoderm, and ectoderm^72–76^, to more advanced free-floating 3D gastrulation models (“3D gastruloids”).

Early works to recapitulate aspects of gastrulation were conducted using mouse stem cells. The first absolute 3D gastruloid was created by van den Brink and colleagues in 2014^69^. Consisting of only 300 mouse ES cells, the EB was cultured in vitro to create a 3D gastruloid, where hallmarks of early mouse embryonic development, such as symmetry breaking and axial organization, were observable under the activation of WNT/β-catenin signaling, which further caused germ layer specification and minor axial elongation upon the addition of specific morphogens such as CHIR99021 and Activin A^77^. The usual 3D gastruloids are cultured through an initial few-hour-to-day EB suspension, whereafter they are then further matured under shaking culture conditions while adding certain morphogens to induce multi-axial organization and a temporal pattern similar to the gene expression of the embryo. Some gastruloids also acquire in vivo patterning integrity by expressing the collinear Hox gene pattern throughout the AP axis^77^.

Through the advancement of the creation of mESC embryonic models, adjustments in the culture conditions of these models enabled more developed structures as well as new discoveries. Although mouse gastruloids effectively mimic embryo-like axial morphogenesis and patterning, they lack regions such as the anterior embryonic region (which includes the brain). To improve this inaccurate depiction of the in vivo embryo, studies have inhibited WNT signaling during the early stages of development to induce gastruloids with anterior neural tissue^68,78^, since elevated expression of genes of the posterior mesoderm was observable with the inducement of WNT signaling^78^. However, WNT signaling is key in breaking axial symmetry in embryonic development, where its set dose induces primitive streak markers and spontaneously breaks AP symmetry. The aforementioned AP axis establishment enables the organization of embryonic tissues. Despite genetic studies that state that this process relies on the embryo’s exposure to extraembryonic spatiotemporally located signals—WNT/β-catenin and Nodal signaling—studies performed with mouse gastruloid models proved that this might not necessarily be the case. Mouse ESC-based gastruloids induced without surrounding extraembryonic tissues showed localized T/Brachyury expression with polarity and extension to one side, suggesting the notion of AP axis development. Thus, this discovery suggested that extraembryonic tissues are not necessarily required for the embryo to undergo self-patterning^69^.

Gastrulation occurs at approximately 16 days post-fertilization (dpf) in human embryos, and in vitro biomimetic models that recapitulate this stage are also achievable by utilizing primed hESCs and hiPSCs, enabling the formation of human gastruloids without the violation of major ethical issues. As in the case of initial murine gastruloid models, human gastruloids were also developed under 2D conditions that portrayed aspects of gastrulation, such as the development of primitive streak-like structures and the three germ layer domains^72,79^.

The development of 3D human gastruloids facilitated the study of human development, as they more accurately depict the in vivo gastrulation process than earlier 2D models. Human PSCs embedded in the matrix, such as by utilizing the commercial extracellular matrix (ECM) formula Matrigel, and cultured under conditions that are supplemented with the right morphogens construct aggregates that undergo symmetry breaking and gene expression patterning^80^. For example, the addition of BMP and WNT agonists was found to be crucial in the establishment of AP polarity and the elongation of the tail bud^81^. Adding extraembryonic compartments resulted in models that more closely resembled the in vivo gastrulating embryo^82^ by mimicking attachment and AP symmetry breaking and further exhibiting human gastrula-specific cells. 3D aggregates that broke symmetry formed extensions along the AP axis with organized germ layers^83,84^. Through the development of the technology of generating human gastruloids, specific gastruloids that addressed parts of the early stages of post-implantation human embryonic development were created, such as the processes of primitive streak formation^80^, neurulation^84–86^, and somitogenesis^87^.

## Late gastruloids modeling somitogenesis and organogenesis

At E7.5 in mice and approximately 20 dpf in humans, the presomitic mesoderm (PSM) condenses and gives rise to somites along the lateral sides of the central neural tube in an anterior-to-posterior fashion. These somites later form structures that act as anchors that hold parts of the embryo, such as the vertebrae, skeletal muscles, cartilage, and dermis. Shortly after somitogenesis has begun, organogenesis is launched, with the heart being the first organ to be generated. Models that mimic somitogenesis, as well as organogenesis, have been created over recent years (specific models are further discussed in the section “Modeling congenital diseases using synthetic embryos”). Segmentation clock waves are observable in mesoderm-based hPSC models (termed “axioloids” or “somatoids”) that mimic segmentation and somitogenesis in the human embryo^88^. Simpler models that recreate oscillatory movements were also generated^89^. Inducing AP symmetry breaking in hPSC models allowed scientists to study the mechanisms underlying human somitogenesis.

Gastruloids have also been able to acquire regions that resemble the gut tube. The formation of the AP and dorsal-ventral axes enables the patterning of the primitive gut tube in mouse gastruloids^90^. More specifically, the anterior foregut, midgut, and hindgut were induced in the gastruloid through the creation of a primordium that covers the overall region of the structure^91^. Furthermore, several gastruloid models, such as trunk-like structure (TLS) gastruloids (using mESCs)^92^ and elongating multi-lineage organized (EMLO) gastruloids (using hiPSCs)^84^, portray gut tube-like structures.

## Development of synthetic whole embryos

Instead of models that recapitulate only a small aspect of the early developing embryo, attempts were made to model its overall characteristics. In mice, the cylindrical shape of the post-implantation embryo is due to the polar TE invagination and actomyosin contractility that give rise to the extraembryonic ectoderm^39^. Stem cell models that resemble these early “egg-cylinders” were generated by combining mESCs with TSCs and incorporating tissues such as those that resemble the extraembryonic ectoderm without recapitulating the blastocyst stage^93^. Adding XEN cells that resemble extraembryonic endoderm cells to these models (ETX or iETX embryos) enabled the production of a visceral endoderm-like epithelium that lines the in vitro conceptus^94,95^. When synthetic embryos were constituents of extraembryonic compartments, they could initiate implantation^95^ and further induce gastrulation, neurulation, and organogenesis^96^.

Several culture techniques that assist in establishing proper embryonic development ex vivo were created, such as circulator systems^97^ and roller culture systems^98^. By taking advantage of these systems, the process of generating whole embryos in vitro was conducted. Due to the aforementioned ISSCR regulation restricting the culture of human embryos to a maximum of 14 days in vitro for research purposes (the “14-day rule”), attempts to create synthetic whole embryos in long-term culture conditions were generally proceeded using mESCs.

To create synthetic whole embryo models, an environment similar to in vivo conditions is needed. With the adaptation of the electronically controlled ex utero roller device that previously cultured a mouse embryo until E11 by the incorporation of the ex utero culture medium (EUCM), which is a mixture of 50% rat serum, 25% Dulbecco’s modified Eagle’s medium (DMEM), and 25% human umbilical cord blood serum^99^, mouse naïve ESCs were cultured until E8.5, which yielded a mouse synthetic whole embryo model that was able to undergo gastrulation and to form organ precursors^100^.

Attempts to create human synthetic whole embryos were also made. For human synthetic whole embryo models, blastoids are currently the most advanced tool, mimicking the entire pre-implantation stage of the human embryo. Although researchers have made attempts to push these blastoids toward the post-implantation stages in vitro, they have not been able to match them with the development of in vivo blastocysts^55,56,60–62^. Recently, there have been strides toward creating a human synthetic whole embryo that can model more advanced developmental processes mimicking post-implantation stages while maintaining the morphological integrity of a natural embryo.

Two groups of scientists have recently reported their attempts at creating human post-implantation embryo models^101,102^. They drew on strategies previously used to create a mouse synthetic whole embryo, which involved differentiating embryonic and extraembryonic cells separately before aggregating them to produce a post-implantation embryo. Weatherbee et al. generated two types of extraembryonic-like cells from hESCs through the overexpression of transcription factors: GATA6 and SRY-box transcription factor 17 (SOX17) for the hypoblast and GATA3 and transcription factor AP-2 gamma (TFAP2C) for TSCs^101^. On the other hand, Oldak et al. utilized ectopic expression of lineage-promoting transgenes to induce the formation of the hypoblast and trophoblast^102^.

Both groups reported human synthetic whole embryo models that replicated the hallmarks of 13-14 dpf human embryos. Weatherbee et al.’s model consisted of structures such as the amnion, extraembryonic mesenchyme, and primordial germ cell-like cells^101^. Oldak et al. reported the formation of the bilaminar disc, epiblast lumenogenesis, amniogenesis, primordial germ cell specification, yolk sac formation, and the expansion of extraembryonic mesoderm in their model^102^. While the creation of human synthetic whole embryo models is expected to continue to evolve, it is essential to closely monitor these developments within the framework of ethical considerations and international agreements.

## Modeling congenital diseases using synthetic embryos

**Fig. 4:**
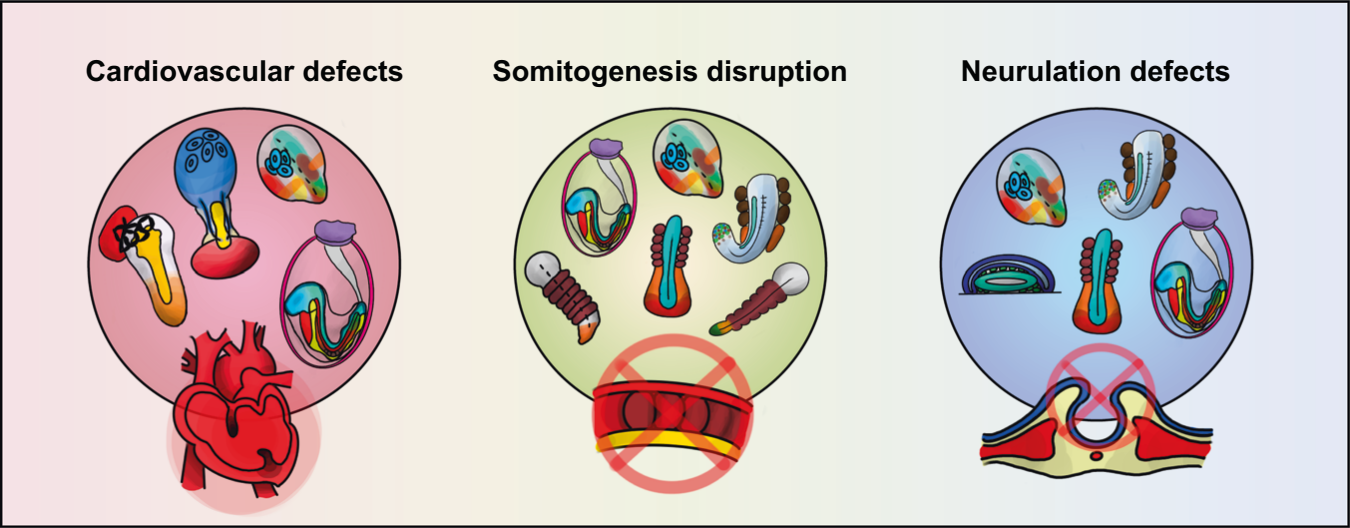
Congenital disease modeling via mouse and human synthetic embryos. Cardiovascular defects (left), diseases associated with somitogenesis disruption (middle), and neurulation defects (right) may be modeled by current gastruloids. Appropriate gastrulation models are depicted in each category.

One of the main advantages of synthetic embryos is their feasibility for modeling diseases that arise during the early stages of embryonic development. Thus, congenital diseases that occur due to initial abnormal development are of interest. Furthermore, because synthetic embryo models such as gastruloids can be easily manipulated to imitate specific stages of development, diseases that occur at a particular event could be observed. Although only a few- considering the subject’s relatively recent advancement into the field, attempts have been made to incorporate synthetic embryoids to model human diseases. This section includes an overview of models that recapitulate key processes of embryonic development with respect to disease modeling (Fig. 4).

Congenital heart disease (CHD) is one of the leading causes of death in newborns^103^. Several models that mimic the heart have been made to recapitulate aspects of cardiogenesis^104–108^; however, to fully understand the dynamics of heart development, observing the organ alone is insufficient. Taking advantage of the gastruloid characteristic of encapsulating multiple cell types of all germ layers, cardiogenesis development is more accurately portrayed by gastruloid models since the formation of the heart necessitates complex interactions such as those among the organ, cardiac progenitor cells, and endothelial cells. Recent studies have mimicked cardiogenesis by manipulating embryo models by incorporating well-known cardiogenic growth factors, such as vascular endothelial growth factor 165 (VEGF-165), FGF2, and ascorbic acid. Rossi et al. captured the process of cardiogenesis via mESC gastruloids, in which the spatiotemporal accuracy was highly conserved compared to its in vivo counterpart^109^. The model incorporates both cardiac progenitors of the first heart field (FHF) and the second heart field (SHF), which anteriorly organize into a cardiac crescent-like region and form tissues that imitate the beating of the heart.

Expanding upon synthetic embryo models that recapitulate only cardiogenesis, new models that include both cardiac muscle and neurons to portray neuro-cardiac lineages have been made. Olmsted and Paluh have added the aspect of cardiogenesis^110^ to the “elongating multi-lineage organized” (EMLO) gastruloids that they had previously generated^111^, creating EMLOC gastruloids that enable the remodeling of both cardiogenesis and neurogenesis to explain the complex interconnected lineages between the processes. Various developmental features that resemble those of the in vivo heart were observed, including the formation of the heart tube, putative outflow tract, ventricular wall, and epicardium, as well as the differentiation of cardiomyocytes. This neuro-cardiac model may offer a platform to provide further understanding of cardiac diseases such as neural-based heart arrhythmia diseases and congenital heart diseases.

Vertebrates acquire segmented structures through a process called somitogenesis. This process occurs in early development when somites, blocks of paraxial mesoderm, form on either side of the neural tube and the notochord in a bilateral position along the AP axis. Presenting phenotypes similar to those of mouse embryos, mouse gastruloids have been offered as appropriate models to study vertebrate segmentation. For instance, genetic alteration of gastruloids yielded phenotypes that matched those of previous knockout mouse embryo models. Veenvliet et al. used mESCs deleted with the T-box transcription factor 6 (*Tbx6*) gene to generate gastruloids (TLSs)^92^. The resulting phenotype was the loss of somites and ectopic neural tubes, which correlates with the in vivo phenomenon of PSM transdifferentiation and ectopic neural tube formation at the expense of somites and PSM. Van den Brink et al. performed a screening test that showed that decreased levels of FGF signaling resulted in the generation of fewer somites, which was similar to the defects observed in *Fgf* mutant mice, and that incorporating 10% Matrigel may induce somitogenesis in gastruloids^71^.

Aberrations in somitogenesis can lead to several diseases, such as vertebral malformations and spinal defects. Specifically, Yamanaka et al. created axioloids that recapitulated human somitogenesis in vitro and applied them to investigate congenital diseases associated with the human spine^88^. They introduced loss-of-function mutations in the segmentation clock gene hes family bHLH transcription factor 7 (HES7) and the marker for the anterior PSM mesoderm posterior bHLH transcription factor 2 (MESP2), which are associated with segmentation defects of the vertebrae (SDV), and generated axioloids with them. Two different HES7 knockout hiPSC lines were used to create axioloids, and their phenotypes were assessed. There were losses of segments and rostro-caudal patterning, as well as the formation of the epithelial somites. Regarding the oscillatory activity of the segmentation clock, Yamanaka and colleagues found a definite loss of HES7 oscillation. In axioloids derived from iPS cell lines introduced with a point mutation in HES7, there were also losses of rostro-caudal patterning, yet these models managed to express normal HES7 oscillation in the tail bud as well as a few somitic mesoderm markers. MESP2 is known to be mutated in patients with SDV. Yamanaka and colleagues also assessed the effects of axioloids with MESP2 knockout. MESP2-knockout axioloids also lacked segments and epithelial somites and exhibited abnormal rostro-caudal patterning, despite their normal elongation. However, MESP2 knockout did not result in the loss of oscillation of HES7. As such, these axioloids serve as models that offer insights into the segmentation development of the human body and a platform to study the pathogenesis of the spine.

Neurulation is a highly orchestrated and complex morphogenetic process in which the neural plate fuses to form the neural tube, the precursor of the brain and spinal cord of the central nervous system. When the process of neurulation is disrupted, severe congenital abnormalities such as neural tube defects (NTDs) arise. Several studies have shown that gastruloids are capable of recapitulating the formation of the neural tube. Libby et al. generated a human neural tube gastruloid that demonstrated aspects of early spinal cord development, such as axial elongation, neuromesodermal progenitor (NMP) cell population maintenance, and neuroepithelial cell generation^81^. With exposure to doses of the WNT agonist CHIR99021, the elongation of hPSC aggregates and the formation of NMP cells could be observed, as well as the presence of the mesoderm and neuroectoderm. Neural tubes were also induced in mouse TLSs created by Veenvliet et al. when cultured in a 5% growth factor-reduced Matrigel condition, as well as by the addition of the WNT agonist CHIR99021 and the BMP signaling inhibitor LDN193189^92^.

Furthermore, neurulation was depicted in partially 3D gastruloids induced by 2D micropatterning by Karzbrun et al., where a neural-tube-like structure was detectable^85^. In this model, Karzbrun and colleagues incorporated three small-molecule inhibitors—the ROCK inhibitor Y-27632, the HSP-90 inhibitor novobiocin, and the NTD-associated drug valproic acid—to test the suitability of these gastruloids for modeling NTDs. The application of the small molecules resulted in morphological abnormalities, including less curvature of the tube and overall folding defects, which indicated that their model may indeed be utilized to represent the neural tube to study defects to some extent.

## Perspectives

Historically, investigating human development has been an ongoing endeavor with significant limitations, including restricted understanding due to the complexity of the process, the lack of appropriate models (not to mention the inaccessibility of human embryos), and ethical concerns. Herein, we have illustrated how the generation of synthetic models of early mammalian development has assisted in overcoming these challenges.

Through the discovery of stem cells and research progress made due to their manipulation, the scope of human embryo experimentation, once available only through direct practice on authentic embryos, became more diverse. By inducing human stem cells to become structures characterized by organized systems, the need for human embryos has diminished, thereby compensating for the inaccessibility of human embryos due to the shortage of samples and ethical issues. Ethical concerns are inevitable; accordingly, regulations have been made by officials such as the ISSCR with recurring discussions among scientists^112^.

Synthetic models do not act merely as models that recapitulate early development; they also provide a platform for which numerous diseases, and their underlying mechanisms, could be further studied. Since these early embryo models may reliably replicate the embryo’s state at certain developmental stages, specific diseases that arise at specific time points may be investigated by recreating a developmentally similar embryonic structure with temporal accuracy. For example, blastoids allow the further study of implantation mechanisms, which then may suggest possible factors associated with implantation failures, and gastruloids may model congenital diseases, including but not limited to those stated above. Current mammalian embryo models are able to recapitulate in vivo embryos of Carnegie stages 1 to 10, in which diseases that arise may be modeled.

As a shared advantage of in vitro structures, synthetic embryo models may be produced in bulk, offering compatibility with systems that require large amounts of samples, such as drug screening and toxicological assays. By using stem cell-based models, researchers have studied the effects of small molecules on mammalian development^113,114^. Furthermore, teratogenicity could be tested through quantifiable gastruloids, which yield statistical robustness, and further applications may be tested, such as species-specific reactions to the molecules^115^.

Nonetheless, a limitation that remains is the total inducement of the synthetic whole embryo in vitro. Several attempts have been made to create synthetic whole embryos, yet results show that there is still a need for further understanding of developmental mechanisms, given the morphological difference of the cultured in vitro embryo with little resemblance to the in vivo embryo and the fact that these synthetic embryos cannot further develop after a set time—not to mention their incompetence to be born.

Furthermore, it should be noted that many in vitro early embryogenesis models representing various developmental stages and dimensions often lack fidelity to in vivo conditions in their cellular composition. Recent work^64^ has revealed that several in vitro models are composed of cells that differ significantly from the transcriptome of their in vivo counterparts. For modes of fidelity validation, the current tools we have to validate the integrity of cell lineages in in vitro models are largely limited to single-cell analyses. At present, single-cell transcriptomics represents the most advanced tool to assess the fidelity of these models. However, even if parts of the in vitro model display gene expression patterns similar to those of its in vivo counterpart, reliance on single-cell transcriptomics alone is insufficient. The number of genes considered per cell in a single-cell analysis ranges from as few as 200 genes to an average of 2000–6000 genes, suggesting that comparisons of similarities between in vitro models and their in vivo counterparts cannot be completely accurate considering the approximate 30,000 genes in the human genome. Much like how the invention of telescopes unveiled previously unseen galaxies and microscopes provided insights into cellular structures, the advancement of tools that govern degrees of accuracy for analyzing differences between in vitro and in vivo embryos will enable us to see new perspectives that lie within the two objects. Thus, there is a clear need for a more sophisticated tool capable of accurately discerning even the subtlest differences. Such innovation would enable the creation of in vitro models that more closely—if not exactly—mirror the in vivo embryo. Further technological improvements would enhance investigations in modeling diseases and pragmatic applications of these models to clinical areas, such as cell-type-specific transplantation in regenerative medicine.

Given that previous works were conducted on less relevant structures via 2D culture and that the field of stem cell and developmental biology has made substantial progress since these advancements in pursuit of generating proper mammalian in vitro embryos that accurately recapitulate developmental stages elicit anticipations of further research improvements and discoveries. The availability of once-seemingly impossible technical applications of lineage-specific stem cell differentiation via these advances will enable further research into discovering new mechanisms that underlie the development of the embryo.
